# Economic Consequences of Surgery for Adhesive Small Bowel Obstruction: A Population-Based Study

**DOI:** 10.1155/2023/1844690

**Published:** 2023-02-25

**Authors:** Thorbjörn Sakari, Sophie Langenskiöld, Filip Sköldberg, Urban Karlbom

**Affiliations:** ^1^Department of Surgery, Gävle Hospital, Gävle, Sweden; ^2^Department of Surgical Sciences, Uppsala University Hospital, Uppsala, Sweden; ^3^Department of Medical Sciences, Uppsala University Hospital, Uppsala, Sweden

## Abstract

**Background and Aims:**

Most patients develop adhesions after abdominal surgery, some will be hospitalized with small bowel obstruction (SBO), and some also require surgery. The operations and follow-up are expensive, but recent data of costs are scarce. The aim of this study was to describe the direct costs of SBO-surgery and follow-up, in a population-based setting. The association between cost of SBO and peri- and postoperative data was also studied.

**Methods:**

In a retrospective cohort study, all patients (*n* = 402) operated for adhesive SBO in Gävleborg and Uppsala counties (2007–2012) were studied. The median follow-up was 8 years. Costs were calculated according to the pricelist of Uppsala University Hospital, Uppsala, Sweden.

**Results:**

Overall total costs were €16.267 million, corresponding to a mean total cost per patient of €40,467 during the studied period. Diffuse adhesions and postoperative complications were associated with increased costs for SBO in a multivariable analysis (*P* < 0.001). Most costs, about €14 million (85%), arouse in conjunction with the SBO-index surgery period. In-hospital stay was the dominating cost, accounting for 70% of the total costs.

**Conclusion:**

Surgery for SBO generates substantial economic burden for healthcare systems. Measures that reduce the incidence of SBO, the frequency of postoperative complication, or the length of stay have the potential to reduce this economic burden. The cost estimates from this study may be valuable for future cost–benefit analyses in intervention studies.

## 1. Introduction

After abdominal surgery, adhesions almost invariably develop and will become symptomatic in a substantial proportion of patients [1]. Clinical manifestations include small bowel obstruction (SBO), female infertility, and abdominal pain. Adhesions can also make future abdominal surgery more complicated [2, 3]. The estimated risk of subsequent surgery for SBO varies with the extent and location of the index procedure (e.g., 1.3% after appendectomy, 5% after colorectal surgery, and up to 14% after proctocolectomy) [4–6]. Apart from suffering of patients, adhesions may lead to substantial costs for the health care systems.

The cost for hospital stays and visits increases over time; in Sweden, it increased 20% during the 5-year period 2012–2017, whereas the Swedish consumer price index rose only by 2.5% in the same time period. Furthermore, there has been a threefold increase in cost for healthcare since 1990 [7].

Previous studies analysing the costs of adhesions [8–12] are heterogeneous in design, and costs estimates vary by a factor of up to almost 4 [9, 13]. A sufficiently sized population-based study could provide representative, detailed, and precise analyses of costs associated with SBO. A detailed knowledge of costs associated with SBO surgery can give valuable data for future interventional studies.

The aim of this descriptive study was to estimate direct costs of surgery and follow-up including readmissions for SBO. A secondary aim was to study costs in relation to peri-and postoperative data.

## 2. Methods

The study included all adult patients operated upon for adhesive SBO in the Uppsala and Gävleborg regions (*n* = 402) during a 5-year period (2007–2012). Adult patients (≥18 years) operated upon for SBO were identified with procedure and diagnostic codes in the medical record systems [14]. In total, there were 618,614 inhabitants in the regions, comprising 6.5% of the Swedish population.

A detailed retrospective analysis of clinical outcome of surgery for the cohort has previously been reported [15]. Demographic data and clinical results from that study are given in Supplementary Tables S1 and S2.

Data on follow-up from medical records have then been re-analysed regarding outpatient visits, emergency visits, radiology, in-hospital stay, surgery, and intensive care unit (ICU)-care and are updated as of February 2022. Patients were followed to death or last note in medical records; follow-up time was calculated from day of index SBO-operation until death or last note in medical records. Patients lost to follow-up (e.g., foreigners) were included only as long as they had any documented contact with the participating hospitals. The study was approved by the local ethical committee at Uppsala University (Registration number 2015/196) and registered atClinicalTrials.gov(NCT03602352/https://clinicaltrials.gov/ct2/show/NCT03602352?cond=Small+Bowel+Obstruction&draw=3&rank=24/).

## 3. Costs

Costs estimates were based on the pricelist of Uppsala University Hospital, Uppsala, Sweden (Supplementary Table S3). Laboratory tests, microbiology, intravenous nutrition, and other medications were included in the daily in-hospital price for surgical ward care. Costs derived from each patient's index admission to the end of follow-up were analysed. Due to missing data of anesthesia time before and after surgery, time of surgery was used as an approximation of the anesthesia time. The cost attributed to the index SBOsurgery included the 30-day postoperative period or the total hospital stay if this was longer than 30 days. Costs generated after the index admission included all hospital re-admissions and outpatient visits related to SBO. Consequently, visits to emergency department with SBO symptoms or abdominal pain without other specific explanation were included as well as SBOrelated visits to the surgical outpatient clinics. Data regarding sick leave from work after hospitalization for SBO were incomplete in medical records so no analysis was performed of the 157 patients of working age (<65 years). Costs are expressed in Euros (1 SEK = 0.107€).

## 4. Statistical Methods

**Figure 1:**
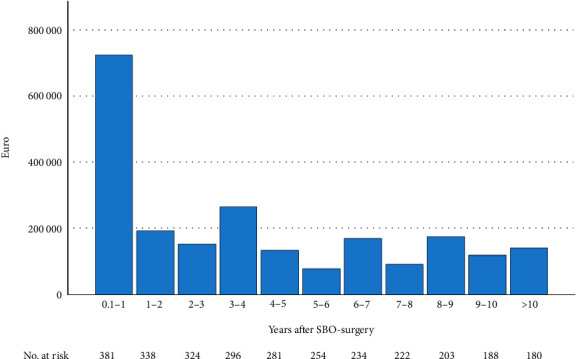
Total costs (€) and numbers at risk each year during the follow-up period after index SBO-surgery.

**Figure 2:**
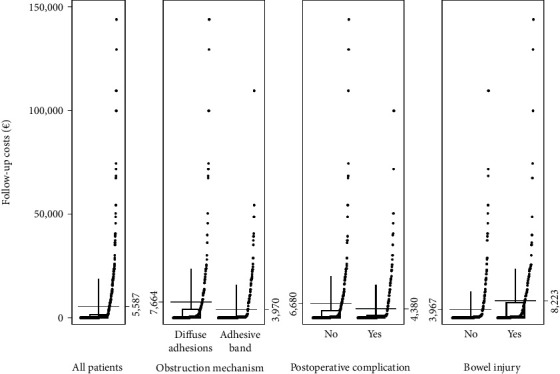
Total costs (€) for index SBO-surgery and the follow-up period. Data are presented for all patients and separately stratified by mechanism of obstruction, bowel injury, and postoperative complication. Numbers on the sides of the graphs represent mean cost per patient.

Distribution of data were assessed with histograms and the Shapiro–Wilks test. As the data had a non-normal distribution, a non-parametric test (Mann–Whitney *U*-test) was used for analysing differences in costs between groups. Means are primarily reported, since these appear most relevant for assessing the economic impact on the health care system. Additionally, medians and inter quartile ranges are reported. A non-parametrical test,Kruskal–Wallis test were used for analysing differences in costs between American Society of Anesthesiologists (ASA) classes. In a multivariable linear regression model, age, gender, ASA class, type of adhesion, the presence/absence of intraoperative bowel injury, and postoperative complications were used as possible predictors. Since the study was purely descriptive, no power calculation was performed. Data were analysed, and Figure 1 was generated using the statistical package SPSS® version 25 for Windows® (SPSS, Chicago, IL, USA), and Figure 2 was generated using the Stata version 17 (StataCorp, College Station, TX, USA). A two-sided *P*-value of <0.05 was considered to be statistically significant.

## 5. Results

During follow-up, 213 of the 402 patients (53%) died. Thirty deaths were possibly caused by SBO, of whom 21 patients died during the initial 30-day postoperative period, and another 9 patients died later on. Later, non-SBO related deaths occurred mainly because of cardiovascular, respiratory, and malignant diseases.

The follow-up was a median of 97 (0–182) months. During this time, 93 patients (23%) had been hospitalized 1–13 times due to SBO, 29 (31%) of them requiring surgery (five patients twice). Among the patients surviving the entire follow-up period (*n* = 189, mean follow-up time 132 months) SBO events were more common with, 51 patients suffering from SBO (27%), 17 of them (33%) required surgery, 3 of these patients twice.

### 5.1. Costs in Conjunction with Index Admission

The total cost for the index admission (including 30 day postoperative period) was €14.022 million, and subsequently, the estimated mean cost per patient was €34,879. Costs were primarily attributed to surgical ward care €9,853,941 (70%), followed by costs of surgery €2,079,743 (15%), intensive care €1,888,161 (13%), and radiology €199,687(1%).

### 5.2. Costs during Follow-Up

Among the 402 patients, 126 patients had scheduled postoperative revisits, and 30 other patients had revisits on patients request related to SBO. One hundred-thirteen visits to the emergency department were noted whereof 43 patients with typical symptoms of SBO and 70 patients with unclear abdominal pain without other explanation. During the study period, 93 patients were hospitalized due to recurrence of SBO, where of 29 required surgery. Totally, the cost of follow-up was €2,246,084. The costs of follow-up were highest the first postoperative year and then decreased to a yearly mean of €152,156 (Figure 1).

Using the full cohort as the denominator, the mean estimated cost during follow-up (including all of the above) per patient was €5,587.

### 5.3. Total Costs

The mean overall cost per patient for the entire study period was €40,467, of which 85% arose in conjunction with the index admission for SBO. Most of the costs €11,701,255 (72%) were related to surgical ward care, followed by costs for operations €2,247,854 (14%), ICU-care €1,906,612 (12%), and radiology €284,611 (2%). Estimates of total costs during the study period were not associated with age, gender, or the hospital of index admission (data not shown). However, in a univariate analysis, patients with higher ASA class had higher total cost but lower costs for follow-up, but those with higher ASA class had significantly shorter follow-up time (Supplementary Table S4).

Nineteen patients (out of 402) had been operated with laparoscopic technique before index SBO surgery, and cost for follow-up and total cost were not significantly different when comparing open and laparoscopic surgery (*P* = 0.995 and *P* = 0.122).

### 5.4. Costs Related to Perioperative Variables

Diffuse adhesions (as opposed to an adhesive band) as the mechanism of obstruction, the occurrence of intraoperative bowel injury, and the occurrence of postoperative complications were associated with increased total cost during the study period (Figure 2; data and statistical parameters available in Supplementary Table S5). Longer operating time and anesthesia, longer in-hospital stay, increased use of radiology, and ICU care contributed to the higher cost in patients with diffuse adhesions. In patients with postoperative complications, cost for operating time and anesthesia, longer in-hospital stay, and increased ICU care were contributing factors, whereas costs for radiology were similar (Supplementary Table S6). In patients where an intraoperative bowel injury occurred, the cost increase could be attributed to operating time and anesthesia, longer in-hospital stay, and increased use of radiology, whereas costs for ICU care were not increased (Supplementary Table S7).

In a multivariable linear regression model, age, gender, ASA-class, obstruction mechanism, intraoperative bowel injury, postoperative complications as independent variables, diffuse adhesions, and postoperative complications were found to be associated with increased total costs during the study period (Table 1).

## 6. Discussion

Our descriptive study provides a large cohort with a detailed assessment of direct costs associated with SBO surgery. The mean cost was estimated to €40,467 per patient including the index SBO operation and follow-up.

The strengths of the study include its population-based design, detailed data, and the degree and length of follow-up. The study also has some limitations, being retrospective and only including patients operated for SBO. Conservatively, managed SBO is common and has been reported to cost almost as much as operative management of SBO patients [8]. However, this study does not account for that, apart from re-occurring SBO during follow-up. Moreover, indirect costs, such as sick leave, could not be assessed. Consequently, overall costs for adhesive SBO are bound to be substantially higher.

The cost estimates presented here are higher than in previous reports [10, 11, 16]. The main cost driver, both for the index admission with SBO surgery and the follow-up period, is in-hospital stay, which is in agreement with earlier studies. A higher price for in-hospital care is a major factor contributing to this difference. It may also be partly due to a general increase in healthcare cost over time [7]. However, most importantly the price for in-hospital care represents the calculated real cost for the treating hospitals. In a broader perspective, adhesions are also associated with female infertility [17] and abdominal pain, for which patients do not seek hospital care, which add to health care costs [18].

Overall mortality among older patients was high, thereby reducing the number of patients at risk for SBO events during follow-up. Still, total costs during follow-up were not affected by age. Patients with higher ASA class had greater total costs, but less costs for follow-up, probably explained by shorter follow-up time due to increased mortality. The mechanism of obstruction, the occurrence of intraoperative bowel injury, and postoperative complications were strongly associated with increased costs in univariate analyses. However, due to co-variation between variables, only diffuse adhesions and complications remained associated with increased costs in multivariable analysis.

Efforts to reduce the clinical and economic burden from SBO surgery could include preventive measures, reducing the incidence of adhesive SBO, measures to optimize the management of SBO, and possibly reducing the recurrence rate of SBO. Laparoscopic surgery has been reported to decrease postoperative SBO; therefore, an increased use of minimally invasive surgery [19–22] is one way to reduce postoperative SBO events. Any reduction in the frequency of complications after abdominal surgery, such as Enhanced Recovery After Surgery (ERAS) programs [23], is also likely to have a positive impact on SBO incidence.

Agents reducing the formation of adhesions may also have a potential to reduce SBO [24]; icodextrin is an example of a liquid anti-adhesion agent, which reaches the entire abdominal cavity and, thereby, possibly could address diffuse adhesions. More data and cost–benefit analyses are needed to evaluate different medical treatments.

In our institutions, all patients without signs of bowel strangulation on clinical evaluation and radiology will be admitted and start with conservative management. Thereafter, if the clinical evaluation shows deterioration or if there is lack of progress in small bowel follow through, surgery will be considered and discussed with the patient. However, further research on the optimal management of SBO, including on whom and when to operate, could also contribute to better management and, thereby, a way of reducing costs [25]. Programs, like the World Health Organization (WHO) checklist [26], for ensuring optimal surgical circumstances during SBO surgery, thereby reducing, for example, the frequency of intraoperative bowel injuries, and implementing protocols for pre- and postoperative care, such as ERAS protocol [23], may also be beneficial. In addition, increased use of laparoscopic SBO surgery in suitable patients, like those with adhesive bands, could decrease costs through less in-hospital stay [27]. The present study provides data on costs that can be particularly useful for the design of studies addressing the economic aspects of these issues.

## 7. Conclusion

In summary, surgery for SBO generates substantial economic burden for healthcare systems. Measures that reduce the incidence of SBO, the frequency of postoperative complication, or the length of stay have the potential to reduce this economic burden. The cost estimates from this study may be valuable for future cost–benefit analyses in intervention studies.

## Figures and Tables

**Table 1:** Multivariable linear regression analysis of total costs (Euro).

Predictor	Estimate (€)	95% Confidence interval (€)	*P*-value
Mechanism	19,529	10,834 to 28,224	<0.001
Complication	19,486	18,621 to 57,080	<0.001
Bowel injury	5,552	−3,313 to 14,417	0.219
Gender	2,012	10,136 to 6,112	0.627
Age	−0.50	−261 to 260	0.997
ASA-class	1,304	−4,784 to 7,366	0.673

## Data Availability

Data supporting this research article are available from the corresponding author or first author on reasonable request.

## Supplementary Materials

Supplementary materials Supplementary Table S1. Demographic data and previous abdominal surgery. Supplementary Table S2. Clinical results from index SBO-surgery and the follow-up period. Supplementary Table S3. Pricelist according to University Hospital, Uppsala. Supplementary Table S4. Costs (€) per patient regarding total and follow-up period stratified by ASA class. Supplementary Table S5. Costs (€) per patient regarding total and follow-up period stratified by mechanism of small bowel obstruction. Supplementary Table S6. Costs (€) per patient regarding total and follow-up period costs related to complications at index surgery. Supplementary Table S7. Costs (€) per patient regarding total and follow-up period costs related to bowel injury at index surgery.
