# Supplementary material for: Economic Consequences of Surgery for Adhesive Small Bowel Obstruction: A Population-Based Study
Source: Gastroenterol Res Pract. 2023 Feb 25;2023:1844690. doi: 10.1155/2023/1844690 (PMC9985498; doi:10.1155/2023/1844690)
Supplement: Supplementary materials — Supplementary Table S1. Demographic data and previous abdominal surgery. Supplementary Table S2. Clinical results from index SBO-surgery and the follow-up period. Supplementary Table S3. Pricelist according to University Hospital, Uppsala. Supplementary Table S4. Costs (€) per patient regarding total and follow-up period stratified by ASA class. Supplementary Table S5. Costs (€) per patient regarding total and follow-up period stratified by mechanism of small bowel obstruction. Supplementary Table S6. Costs (€) per patient regarding total and follow-up period costs related to complications at index surgery. Supplementary Table S7. Costs (€) per patient regarding total and follow-up period costs related to bowel injury at index surgery. [file 1844690.f1.docx]

**Supplement Table S1**. Demographic data and previous abdominal surgery.

|  | **All patients** (n=402) |
| --- | --- |
| Gender M/F | 162/240 |
| Age, years, median (range) | 70 (18-97) |
| ASA 1- 2 § | 204 (51%) |
| ASA 3- 4 | 193 (48%) |
| Cardiovascular disease | 202 (50%) |
| COPD # | 45 (11%) |
| Diabetes | 36 (9%) |
| Immunosuppression | 25 (6%) |
|  |  |
| No. of previous abdominal operations |  |
| 0 | 50 (12%) |
| 1 | 189 (47%) |
| 2 | 81 (20%) |
| 3 | 60 (15%) |
| ≥4 | 22 (6%) |
|  |  |
| Type of previous procedure ¤ |  |
| Appendectomy | 115 (29%) |
| Colorectal | 110 (27%) |
| Gynaecological | 106 (26%) |
| Upper GI | 104 (26%) |
| Surgery for SBO | 30 (7%) |

§ 5 missing diagnoses, # chronic obstructive pulmonary disease, ¤ Many patients with several previous operations, hence sum > 100%

**Supplement Table S2.** Clinical results from index SBO-surgery and the follow-up period.

|  | **All patients** (n=402) |
| --- | --- |
| No. previous operations # | 1.6±1.1 |
| Time from admission to surgery (days) # | 3.4±6.9 |
|  |  |
| Bleeding (ml) # | 189± 339 |
| Operation time (min) # | 96± 63 |
| Bowel injury | 153 (38%) |
| Any bowel resection | 139 (35%) |
|  |  |
| Early complications | 191 (48%) |
| Anastomotic leak (n=139) | 4 (3%) |
| Pneumonia | 32 (8%) |
| Arrhythmia | 21 (5%) |
| Urinary tract infection | 32 (8%) |
|  |  |
| Postoperative stay (days) # | 11±11 |
| Length of stay (days) # | 15±13 |
|  |  |
| Late complications | 191 (48%) |
| SBO- admission | 93 (23%) |
| No. times admission for SBO (range) | 0-13 |
| SBO- surgery | 29 (7%) |
| Follow-up time (months) # | 90±59 |
| Deceased at follow-up | 213 (53%) |

# Mean ± standard deviation

**Supplement Table S3**. Pricelist according to University Hospital, Uppsala.

|  | **SEK §** | **Euro #** | **USD ¤** |
| --- | --- | --- | --- |
| **In-hospital stay** |  |  |  |
| Day 1 | 15 929 | 1 664 | 1 750 |
| Day 2- | 15 409 | 1 610 | 1 693 |
| **ICU per day** | 57 480 | 6 006 | 6 316 |
|  |  |  |  |
| **Surgery** |  |  |  |
| Operation, per minute | 345 | 37 | 38 |
| Anaesthesia, per minute | 110 | 11.5 | 12.1 |
|  |  |  |  |
| **Radiology** |  |  |  |
| Plain abdominal view, office hours | 1 107 | 116 | 122 |
| Plain abdominal view, on call | 1 770 | 185 | 195 |
| CT-overview, office hours | 1 320 | 138 | 145 |
| CT-overview, on call | 2 099 | 219 | 231 |
| CT with iv contrast, office hours | 1 526 | 159 | 168 |
| CT with iv contrast, on call | 2 426 | 254 | 267 |
| Small bowel follow-through, office hours | 2 225 | 232 | 245 |
| Small bowel follow-through, on call | 3 555 | 371 | 391 |
|  |  |  |  |
| **Outpatient visits** |  |  |  |
| Outpatient visit surgery, planned or patient request | 4 657 | 487 | 512 |
| Outpatient visit emergency ward | 4 094 | 428 | 450 |

§ SEK=Swedish krona, # Euro=Exchange rate at 20161230, ¤ US Dollar=Exchange rate at 20161230

**Supplement Table S4.** Costs (€) per patient regarding total and follow-up period stratified by ASA class.

|  | **ASA class 1 n=52** | **ASA class 2 n=152** | **ASA class 3 n=170** | **ASA class 4 n=23** | **p-value** |
| --- | --- | --- | --- | --- | --- |
|  | mean/median (IQR) | mean/median (IQR) | mean/median (IQR) | mean/median (IQR) |  |
| **Total costs (€)** |  |  |  |  |  |
| In-hospital stay | 21 241/14 895 (16 488) | 28 396/19 841 (19 841) | 32 888/24 787 (19 827) | 25 726/23 138 (9 040) | <0.001 |
| Radiology | 766/533 (494) | 688/520 (583) | 720/605 (559) | 622/605 (623) | 0.546 |
| Operation and anaesthesia | 5 297/3 724 (4 502) | 5 802/4 382 (4 321) | 5 620/4 613 (3 968) | 4 892/4 138 (4 041) | 0.792 |
| ICU-stay | 237/0 (0) | 4249/0 (0) | 4776/0 (0) | 18 986/0 (12 301) | <0.001 |
| Revisits | 306/438 (498) | 315/0 (498) | 320/0 (498) | 233/0 (498) | 0.167 |
|  |  |  |  |  |  |
| Sum of total costs | 27 845/22 894 (19 141) | 39 450/26 836 (22 625) | 44 324/32 276 (29 831) | 50 459/33 114 (23 131) | <0.001 |
|  |  |  |  |  |  |
| **Costs during follow-up (€)** |  |  |  |  |  |
| In-hospital stay | 4 285/0 (0) | 5 258/0 (0) | 4 687/0 (0) | 581/0 (0) | 0.571 |
| Radiology | 285/0 (0) | 225/0 (0) | 194/0 (0) | 79/0 (0) | 0.729 |
| Operation and anaesthesia | 389/0 (0) | 654/0 (0) | 285/0 (0) | 0/0 (0) | 0.191 |
| ICU-stay | 0/0 (0) | 0/0 (0) | 109/0 (0) | 0/0 (0) | 0.721 |
| Revisits | 306/438 (498) | 315/0 (498) | 320/0 (498) | 233/0 (498) | 0.167 |
|  |  |  |  |  |  |
| Sum of follow-up costs | 5 265/498 (623) | 6 452/438 (1 559) | 5 586/0 (1 325) | 893/0 (498) | 0.574 |
| Follow-up time (months) | 122/141 (44) | 114/128 (68) | 65/53 (96) | 43/11 (57) | <0.001 |

**Supplement Table S5.** Costs (€) per patient regarding total and follow-up period stratified by mechanism of small bowel obstruction.

|  | **Diffuse adhesions (n=176)** | **Adhesive band (n=226)** | **p-value** |
| --- | --- | --- | --- |
|  | mean/median  (IQR) | mean/median  (IQR) |  |
| **Total costs (€)** |  |  |  |
| In-hospital stay | 37 926/26 464  (26 806) | 22 240/19 841  (16 488) | <0.001 |
| Radiology | 843/612  (510) | 603/522  (500) | <0.001 |
| Operation and anaesthesia | 7 046/5 404  (4 686) | 4 459/3 408  (3 554) | <0.001 |
| ICU-stay | 7 478/0  (0) | 2 612/0  (0) | 0.335 |
| Revisits | 357/0  (498) | 285/0  (498) | 0.215 |
|  |  |  |  |
| Sum of total costs | 53 650/33 758  (37 658) | 30 200/24 244  (19 410) | <0.001 |
|  |  |  |  |
| **Costs during follow-up (€)** |  |  |  |
| In-hospital stay | 6 422/0  (3 353) | 3 172/0  (0) | 0.027 |
| Radiology | 300/0  (163) | 142/0  (0) | 0.037 |
| Operation and anaesthesia | 584/0  (0) | 289/0  (0) | 0.843 |
| ICU-stay | 0/0  (0) | 82/0  (0) | 0.380 |
| Revisits | 357/0  (498) | 285/0  (498) | 0.215 |
|  |  |  |  |
| Sum of follow-up costs | 7 664/498  (4 038) | 3 970/0  (498) | 0.014 |

**Supplement Table S6.** Costs (€) per patient regarding total and follow-up period costs related to complications at index surgery.

|  | **Any complication (n=191)** | **No complication (n=211)** | **p-value** |
| --- | --- | --- | --- |
|  | mean/median  (IQR) | mean/median  (IQR) |  |
| **Total costs (€)** |  |  |  |
| In-hospital stay | 34 582/24 787  (24 731) | 24 152/18 248  (14 839) | <0.001 |
| Radiology | 677/544  (526) | 736/544  (583) | 0.782 |
| Operation and anaesthesia | 6 493/5 063  (4 357) | 4 776/3 797  (3 457) | <0.001 |
| ICU-stay | 9 789/0  (6 150) | 175/0  (0) | <0.001 |
| Revisits | 387/0  (498) | 253/0  (498) | 0.573 |
|  |  |  |  |
| Sum of total costs | 51 928/34 389  (36 403) | 30 091/23 960  (17 372) | <0.001 |
|  |  |  |  |
| **Costs during follow-up (€)** |  |  |  |
| In-hospital stay | 3 563/0  (0) | 5 530/0  (3 353) | 0.070 |
| Radiology | 153/0  (0) | 264/0  (141) | 0.069 |
| Operation and anaesthesia | 277/0  (0) | 546/0  (0) | 0.139 |
| ICU-stay | 0/0  (0) | 87/0  (0) | 0.344 |
| Revisits | 387/0  (498) | 253/0  (498) | 0.573 |
|  |  |  |  |
| Sum of follow-up costs | 4 380/0  (936) | 6 680/438  (3 505) | 0.202 |

**Supplement Table S7.** Costs (€) per patient regarding total and follow-up period costs related to bowel injury at index surgery.

|  | **Bowel injury (n=153)** | **No Bowel injury (n=249)** | **p-value** |
| --- | --- | --- | --- |
|  | mean/median  (IQR) | mean/median  (IQR) |  |
| **Total costs (€)** |  |  |  |
| In-hospital stay | 35 061/24 843  (24 731) | 25 450/19 841  (16 488) | <0.001 |
| Radiology | 849/605  (618) | 621/522  (500) | <0.001 |
| Operation and anaesthesia | 7 408/5 842  (5 283) | 4 476/3 651  (3 213) | <0.001 |
| ICU-stay | 5 668/0  (0) | 4 174/0  (0) | 0.708 |
| Revisits | 377/0  (498) | 280/0  (498) | 0.002 |
|  |  |  |  |
| Sum of total costs | 49 362/33 562  (28 918) | 35 001/25 527  (21 599) | <0.001 |
|  |  |  |  |
| **Costs during follow-up (€)** |  |  |  |
| In-hospital stay | 6 922/0  (6 651) | 3 166/0  (0) | <0.001 |
| Radiology | 321/0 (327) | 144/0  (0) | <0.001 |
| Operation and anaesthesia | 604/0  (0) | 304/0  (0) | 0.953 |
| ICU | 0/0  (0) | 74/0  (0) | 0.436 |
| Revisits | 377/0  (498) | 279/0  (498) | 0.002 |
|  |  |  |  |
| Sum of follow-up costs | 8 223/498  (7 219) | 3 967/0  (498) | <0.001 |
